# Ageing exacerbates damage of systemic and salivary neutrophils from patients presenting *Candida*-related denture stomatitis

**DOI:** 10.1186/1742-4933-6-3

**Published:** 2009-03-28

**Authors:** Thaís Helena Gasparoto, Narciso Almeida Vieira, Vinicius Carvalho Porto, Ana Paula Campanelli, Vanessa Soares Lara

**Affiliations:** 1 Department of Biological Sciences (Microbiology and Immunology), Bauru Dental School, Bauru, SP, Brazil; 2 University of São Paulo (USP), Bauru, SP, Brazil; 3 Clinical Analysis Laboratory of the Hospital for Rehabilitation of Craniofacial Anomalies, University of São Paulo (USP), Bauru, SP, Brazil; 4 Department of Prosthodontics, University of São Paulo (USP), Bauru, SP, Brazil; 5 Department of Stomatology (Oral Pathology), Bauru Dental School, Bauru, SP, Brazil

## Abstract

**Background:**

Ageing leads to a decline in the function of the immune system, increasing the body's susceptibility to infections through the impairment of T-cells, macrophages, neutrophils and dendritic cells Denture stomatitis is a primary oral disease affecting elderly denture wearers. The major etiologic factor involved in this pathology is the infection by *Candida albicans*, an opportunistic pathogen that causes local and disseminated diseases in immunosuppressed humans. Neutrophils play a critical role in the immune response against *C. albicans *and are continually present in the salivary fluid and in the blood. The aim of this study was to determine ageing-related changes in salivary and blood neutrophils and their potential implications in *Candida*-related denture stomatitis.

**Results:**

Our results showed a lower number of neutrophils in the saliva from patients presenting *Candida*-related denture stomatitis in comparison to their matched controls. Furthermore, fewer neutrophils were isolated from the saliva of aged control individuals in comparison to matched younger subjects. CXCR1, CD62L and CD11b expression were significantly greater on systemic neutrophils from younger control individuals. Elderly individuals showed more apoptotic salivary neutrophils and lower GM-CSF levels than younger ones, regardless of the occurrence of *Candida *infection. On the other hand, CXCL-8 concentrations were higher in the saliva from elderly individuals. Besides, TNF-α was detected at elevated levels in the saliva from infected elderly subjects. Salivary neutrophils from elderly and young patients presented impaired phagocytic activity against *C. albicans*. However, just systemic neutrophils from elderly showed decreased phagocytosis when compared to the younger ones, regardless of the occurrence of infection. In addition, neutrophils from aged individuals and young patients presented low fungicidal activity.

**Conclusion:**

The data suggests that the *Candida *related-denture stomatitis is associated to neutrophils function deficiency, and ageing drastically appears to alter important characteristics of such cells, facilitating the establishment of this infection.

## Background

Elderly people suffer higher rates of morbidity and mortality from infectious diseases than younger adults [1]. The most common oral mucosal lesion in elderly people is denture stomatitis (D.S.) [2-6], an inflammatory condition that affects denture wearers worldwide, and is associated with the presence of yeasts, especially *Candida albicans *[7-10].

*C. albicans *is an opportunist microorganism that shifts from commensal to pathogenic status in human mucosa. Such shift depends on environmental predisposing factors such as diabetes, antibiotic therapies, and immunodepression [11,12]. Immune response mechanisms are responsible for controlling the establishment of *Candida*-related infection on oral mucosa [13]. Neutrophils are considered important antifungal cells; they are early recruited to sites of infection and are able to destroy the pathogen by both phagocytosis and production of reactive oxygen species. Their role in such infections is evident since neutropenia is associated with systemic candidiasis [14-16]. Moreover, neutrophils play a key role in the host defense against localized *C. albicans *infections [17].

The function of salivary and blood neutrophils against oral pathogens, including *C. albicans*, has been widely described. Neutrophils influence the establishment of oral diseases [18-21], and disorders involving such cells have been broadly related to recurrent bacterial or fungal oral infections [22]. Although neutrophils are considered to participate in the acute response against pathogens in many tissues, their influx into the oral cavity occurs at any time. Such cells are chemoattracted by factors present in the oral environment such as microorganisms, toxins, chemokines and cellular degradation products. Alterations in the number of neutrophils, and the suppression of their function, such as those observed in systemic lupus erythematosus, have been shown to predispose individuals to oral diseases [23-28]. Considering this context, quantitative or qualitative changes in neutrophils could result in a higher susceptibility to denture stomatitis. Moreover, among the immunological alterations observed in senescence, neutrophils become functionally impaired especially when challenged by infectious agents [10,29-31]. Neutrophils presenting impaired microbicidal capacity against *C. albicans *as well as a significant decrease in the production of reactive oxygen species, even when stimulated by granulocyte-macrophage colony-stimulating factor (GM-CSF), have been shown to occur in the elderly [32-34]. However, neutrophils from aged humans have shown different reactions for various stimuli [35]. Besides, leukocytes from elderly subjects present alterations in cytokine production after microorganisms challenge, generating high IL-10 and IL-4 as well as elevated IL-8 (CXCL8) and TNF-α levels [36].

Aiming at understanding the defense mechanisms that influence denture stomatitis in elderly patients, our study evaluated the number of cells, apoptosis occurrence and *in vitro *phagocytic function of blood and salivary neutrophils. Neutrophils from individuals with 60–85 years old presenting or not *Candida *associated-denture stomatitis were evaluated in the same conditions in comparison to those from younger people (20–50 years old). Cytokines levels in the saliva from these individuals were determined. Besides, CXCR1, CD62L and CD11B expression levels were evaluated on blood neutrophils.

## Results

### Salivary neutrophils counting are reduced in patients with *Candida*-related denture stomatitis as well as in elderly control individuals

**Figure 1:**
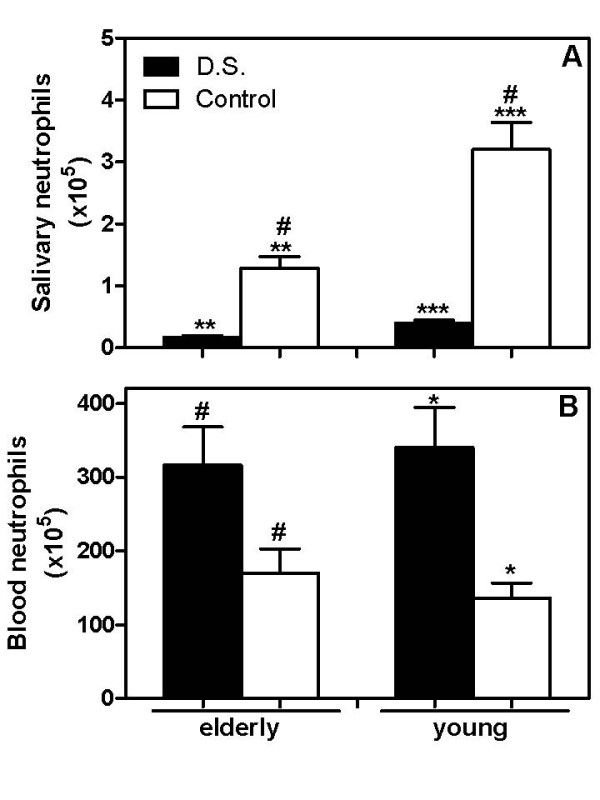
**Number of neutrophils isolated from the saliva and blood of patients with *Candida*-related denture stomatitis (D.S.) and controls**. Saliva and blood samples from diseased (closed bars) and control (open bars) elderly and young groups were obtained as described in the Material and Methods section. The salivary (A) and blood neutrophils (B) were evidenced through staining with Türk's solution and the collected total number determined after counting in a Neubauer chamber. The results are expressed as the mean ± SEM for elderly (*n *= 14) and young (*n *= 14) patients or aged (*n *= 14) and young (*n *= 14) control subjects tested individually. The data were analyzed by one-way ANOVA followed by Tukey's test and the value of *P *was considered significant when < 0.01. Equal symbols indicate significant difference between the groups.

Our results demonstrate that the number of neutrophils obtained from the saliva of elderly D.S. was significantly lower than that of the elderly control (0.2 ± 0.03 × 10^5 ^and 1.3 ± 0.2 × 10^5^, respectively, Figure 1A). Similarly, young individuals with D.S. showed a lower number of salivary neutrophils in comparison to the young control (0.4 ± 0.06 × 10^5 ^and 3.2 ± 0.4 × 10^5^, Figure 1A). Interestingly, young control subjects showed a significantly higher neutrophil counting than elderly controls (*p *= 0.0015).

Although the number of neutrophils isolated from the saliva of patients with D.S. was lower when compared to the respective control groups, the number of neutrophils obtained from the peripheral blood of subjects with denture stomatitis was higher than the respective controls (316.2 ± 51.9 × 10^5 ^– elderly D.S. and 176.3 ± 35.4 × 10^5 ^– elderly control; 305 ± 63.7 × 10^5 ^– young D.S. and 136 ± 21.1 × 10^5 ^– young control, Figure 1B).

### Expression of CXCR1, CD62L and CD11b on blood neutrophils from impaired patients (D.S.) and elderly controls was diminished

**Figure 2:**
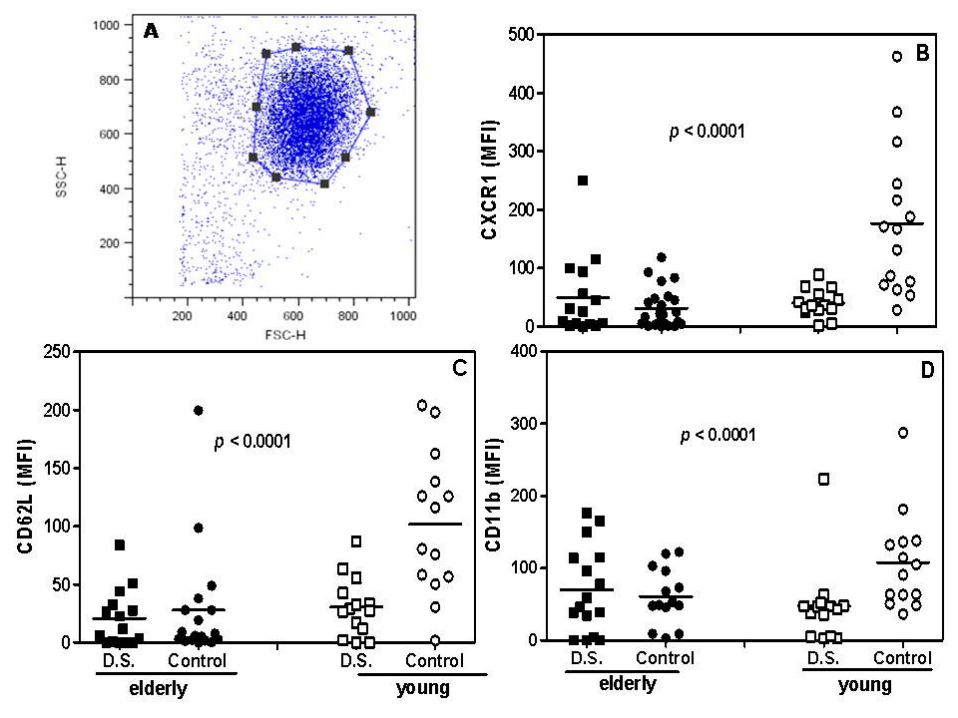
**Detection of CXCR1, CD62L and CD11b on blood neutrophil**. Peripheral blood was obtained from diseased (black and white squares) and control (black and white circles) elderly and young groups and granulocytes were purified as described in the Material and Methods section. The cells were gated on neutrophils (representative picture, 2A) via their forward (FSC) and side scatter (SSC) properties. Mean fluorescence intensity (MFI) of B, CXCR1, C, CD62L, and D, CD11b from all groups was evaluated by flow cytometry. The results are expressed as the mean of MFI ± SEM for elderly (*n *= 14) and young (*n *= 14) patients or aged (*n *= 14) and young (*n *= 14) control subjects tested individually. The data were analyzed by unpaired t-test and the value of *P *was considered significant when <0.05 for young control compared to patients and elderly control.

In order to understand why a lower number of salivary neutrophils was found in elderly and young individuals carrying the disease, as well as in aged controls, we studied the expression of molecules involved in the chemotaxis and diapedesis of neutrophils from the blood of impaired patients and healthy volunteers. Our results showed a significantly lower expression of CXCR1 and CD62L on neutrophils from patients with *Candida*-related denture stomatitis than from young controls. Curiously, blood neutrophils of aged subjects not affected by the disease also expressed lesser amounts of CXCR1 and CD62L than matched young subjects (Figure 2B and 2C). Also, young controls presented a significant higher expression of CD11b on blood neutrophils than all other groups (Figure 2D).

### Salivary neutrophils from aged subjects presented a higher rate of apoptosis

**Figure 3:**
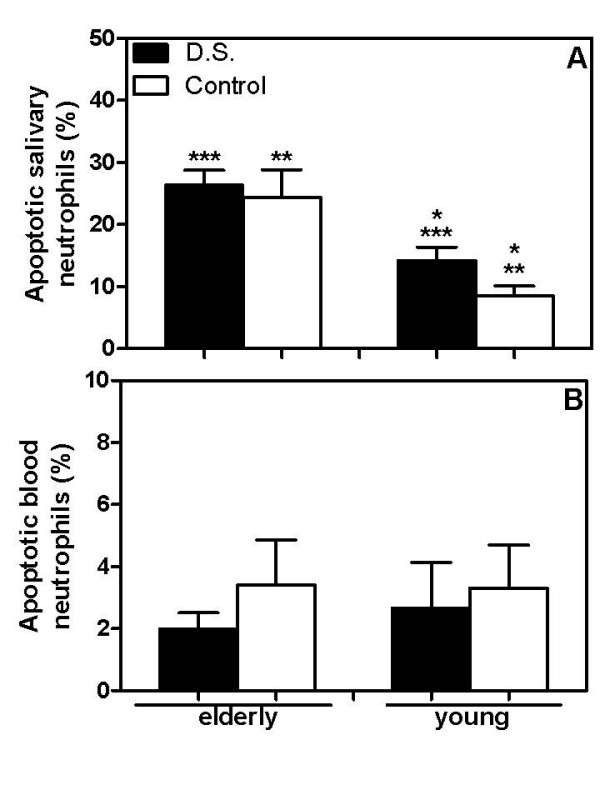
**Apoptosis of salivary and blood neutrophils from patients with *Candida*-related denture stomatitis and controls**. Salivary (A) and blood neutrophils (B) from diseased (closed bars) and control (open bars) elderly and young groups were isolated and, immediately, their viability was analyzed by positivity to annexin V-FITC and propidium iodide. The cells were analyzed by fluorescence microscopy and the percent of apoptotic cells calculated by the proportion of positive neutrophil (green and/or red cells) in relation to total neutrophils. Results are expressed as the mean ± SEM from each group, elderly (*n *= 14) and young (*n *= 14) patients or aged (*n *= 14) and young (*n *= 14) control subjects tested individually. The results were evaluated by one-way ANOVA followed by Tukey's test and the value of *P *was considered significant when < 0.01. Equal symbols indicate significant difference between the groups.

Before performing the assays, the viability of salivary and blood neutrophils was analyzed. Our data showed a significantly higher rate of salivary apoptotic neutrophils in elderly groups (26.3 ± 2.4% – elderly D.S. and 24.4 ± 4.3%-elderly control, Figure 3A) than those from the respective young groups. While elderly groups showed the same rate of apoptotic neutrophils in the saliva, young D.S. had a higher rate of apoptotic neutrophils in the saliva than the matched controls (14.2 ± 2.2% – young D.S. and 8.5 ± 1.6% – young control, *p *= 0.04, Figure 3A). Although salivary neutrophils were apoptotic, blood neutrophils from aged subjects were as viable as those from the young ones, regardless of the disease (from 2 ± 0.5% to 3.4 ± 1.5%, Figure 3B).

### GM-CSF levels were lower in the saliva from aged subjects than from matched young subjects

**Figure 4:**
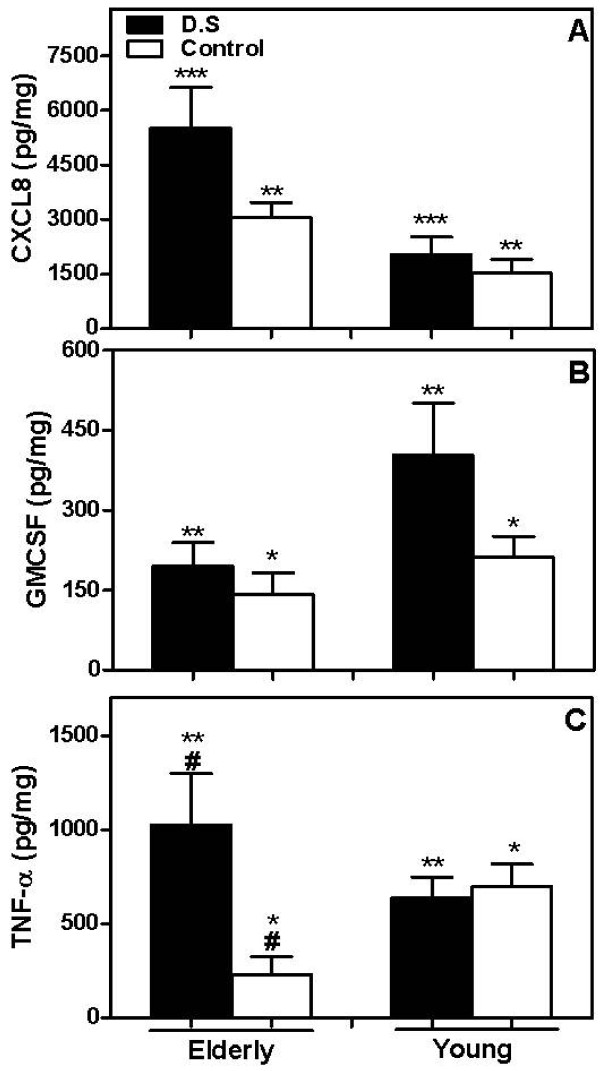
**Detection of the cytokines in the saliva from patients with *Candida*-related denture stomatitis and controls**. Whole saliva samples from each donor were collected and cytokines (CXCL8, GM-CSF and TNF-α) were quantified by ELISA. The mean concentration ± SEM for CXCL8 (A), GM-CSF (B) and TNF-α (C) normalized to total protein in the sample saliva from diseased (closed bars) and control (open bars) elderly and young groups are shown. Data shown are the mean of triplicate values from a single experiment representative of two performed experiments for each donor. The results were evaluated by one-way ANOVA followed by Tukey's test and the value of *P *was considered significant when < 0.01. Equal symbols indicate significant difference between the groups.

Due to the different rate of viability of salivary neutrophils from aged people, the levels of cytokines known to affect neutrophil apoptosis, CXCL8, GM-CSF, and TNF-α were evaluated in the whole saliva from the subjects. CXCL8 was significantly elevated in the saliva from elderly patients and controls (5521 ± 1110 pg/mg and 3058 ± 410.5 pg/mg) when compared to young individuals, presenting or not D.S. (2025 ± 499.8 pg/mg and 1523 ± 391.3 pg/mg respectively, Figure 4A). In addition, elderly D.S. had higher levels of CXCL8 than matched controls (*p *= 0.0002). However, GM-CSF concentrations were lower in the saliva from elderly individuals, regardless of D.S. occurrence (194.9 ± 44.4 pg/mg, and 145.2 ± 40.6 pg/mg in D.S. or Control groups, respectively, Figure 4B). Although elderly D.S. and control groups did not exhibit differences, young D.S. presented significantly elevated concentration of GM-CSF (402 ± 98.8 pg/mg, Figure 4B) in comparison with matched controls (211 ± 39.4 pg/mg, *p *= 0.03).

We detected an increase of the TNF-α level in the saliva from elderly D.S. group (1026 ± 273.1 pg/mg) in comparison with its matched control (229.5 ± 95.8 pg/mg, Figure 4C). Although elderly D.S. displayed higher TNF-α level than young D.S. (638.3 ± 110.8 pg/mg), elderly controls had lower levels of TNF-α than young ones (696.7 ± 122.1 pg/mg, *p *= 0.003, Figure 4C). No differences were detected in TNF-α level when we compared young groups.

### Phagocytosis of salivary neutrophils from patients with *Candida*-related denture stomatitis and aged control was diminished. However, only blood neutrophils from the elderly have impaired phagocytic function

**Figure 5:**
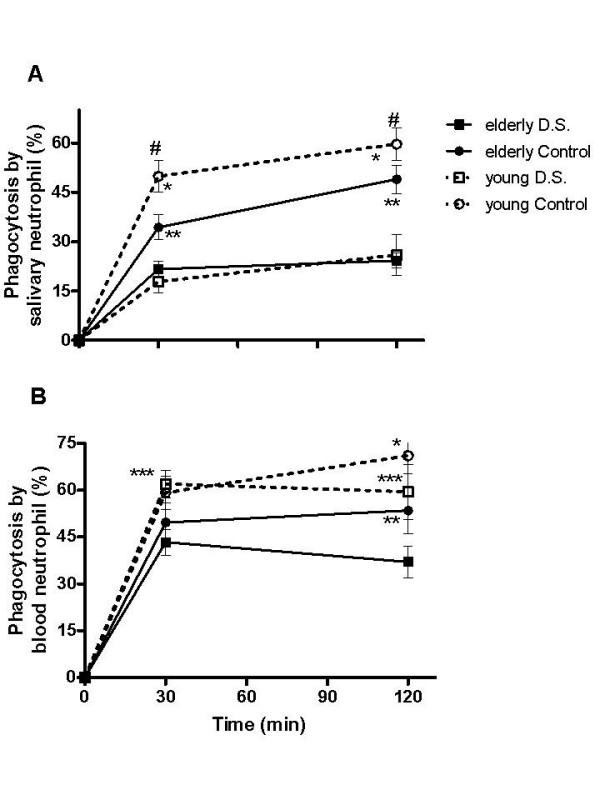
**Phagocytosis of viable *Candida albicans *by salivary and blood neutrophils from patients with *Candida*-wrelated denture stomatitis and controls**. Saliva and blood samples from diseased (black and white squares) and control (black and white circles) elderly and young groups were collected and purified neutrophils were challenged with viable blastoconidia of *C. albicans *ATCC 10231 that had been opsonized with human serum (at a ratio of 1:1 of opsonized yeast: viable neutrophil). After 30 and 120 min, percentage of salivary (A) and blood neutrophils (B) phagocytosing *C. albicans *was assessed through staining with acridine orange and counting of 10 random fields. Results are expressed as the mean ± SEM from each donor analyzed individually. The data were analyzed by one-way ANOVA followed by Tukey's test and the difference was considered significant when *P *< 0.05. Differences are indicated by * young and elderly control groups; ** D.S. and control elderly groups; *** D.S. elderly and D.S. young groups and ^#^D.S. and control young groups.

The ingestion of viable *C. albicans *was determined in purified salivary and blood neutrophils. The phagocytic activity against *C. albicans *by salivary neutrophils obtained from elderly D.S. was significantly lower when compared to the elderly controls (21.7 ± 2.4% and 34.4 ± 3.8%, respectively, Figure 5A), and this low phagocytic activity remained up to 120 min (24.3 ± 2.3%). Nevertheless, significant differences were observed between the phagocytic activity of salivary neutrophils from elderly and young controls at 120 min of the assay (Figure 1A, *p *< 0.01). At 30 min, salivary neutrophils from young controls were able to ingest *C. albicans *more efficiently than those neutrophils from young patients (49.9 ± 4.7% and 17.9 ± 3.5% respectively, Figure 5A). Although at 120 min of the assay the phagocytic activity of salivary neutrophils from young patients raised to 26 ± 6.2% (Figure 5A), this value was significantly lower than that of young controls (59.7 ± 4.9%). Therefore, salivary neutrophils from young and aged patients, and elderly control individuals showed reduced phagocytic activity.

In order to provide additional information on the role of neutrophils in denture stomatitis, besides the influence of ageing in the establishment of this disease, the phagocytic activity of systemic neutrophils was analyzed. At both 30 and 120 min evaluation times, peripheral blood neutrophils from elderly D.S. presented a significantly lower phagocytic activity (43.4 ± 4.1% and 37.1 ± 5.1%, respectively) than young D.S (62 ± 4.4% and 59.5 ± 8.8%, Figure 5B). In the elderly controls, a reduction in *Candida *phagocytosis was observed only after 120 min (53.5 ± 7.5%, Figure 5B). Although at 120 min of the assay the phagocytic activity of blood neutrophils from elderly controls raised, this value was significantly lower than that of young controls (71.1 ± 5.9%, Figure 5B). Taken together, our results demonstrated that systemic neutrophils from elderly groups had an impaired phagocytic function for *C. albicans *in comparison with the neutrophils from matched younger groups.

### Salivary and blood neutrophils from diseased patients and elderly controls killed significantly lesser *C. albicans *cells than those from younger controls

**Figure 6:**
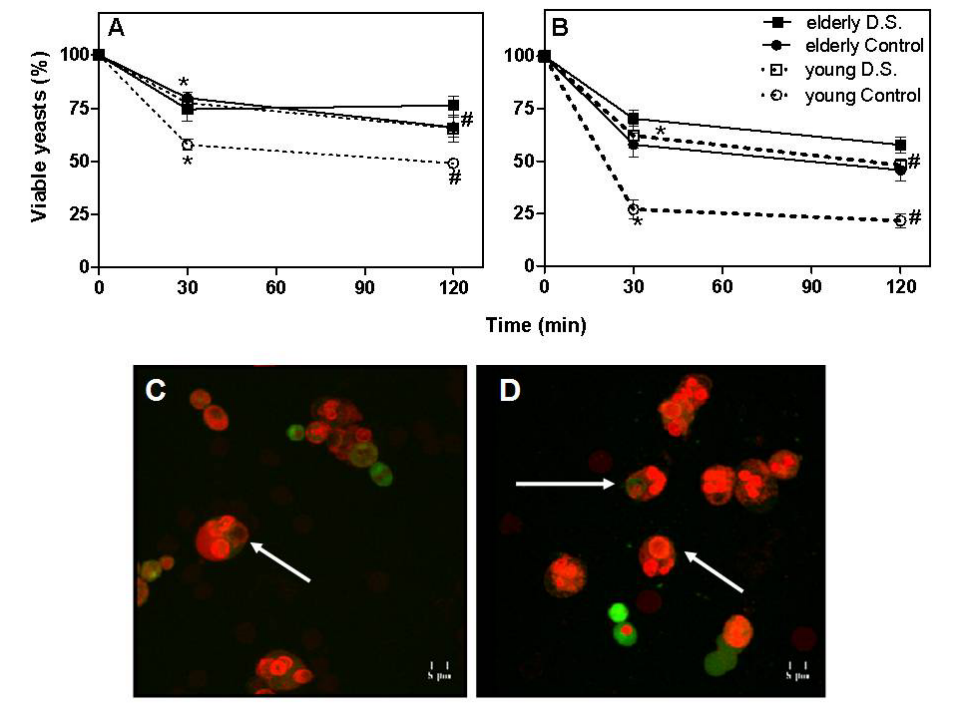
**Viability of phagocytosed yeast forms of *C. albicans *by salivary and blood neutrophils from patients with denture stomatitis and controls**. Saliva and blood samples from diseased (black and white squares) and control (black and white circles) elderly and young groups were collected and purified neutrophils were challenged with viable blastoconidia of *C. albicans *ATCC 10231 that had been opsonized (as described in the Material and Methods section). After 30 and 120 min, the percentage of salivary (A) and blood neutrophils (B) containing viable yeasts was determined through staining with acridine orange and counting of 10 random fields. The yeast cells were considered viable when they exhibited green coloration and dead when exhibited orange coloration. Results are expressed as the mean ± SEM from each group, elderly (*n *= 14) and young (*n *= 14) patients or aged (*n *= 14) and young (*n *= 14) control subjects were analyzed individually. The results were evaluated by one-way ANOVA followed by Tukey's test and the value of *P *was considered significant when < 0.01. Equal symbols indicate significant difference between the groups. Representative fluorescence micrographs of neutrophils phagocytosing *C. albicans *stained by acridine orange are demonstrated in C and D. The arrows label the green viable *C. albicans *and the orange dead *C. albicans *yeasts.

After demonstrating impaired phagocytic function of the salivary neutrophils from D.S. patients, the death of blastospores that were ingested by salivary and blood neutrophils was assessed. When it comes to salivary neutrophils, young controls neutralized more ingested blastoconidia than all the evaluated groups because they had less viable internalized *C. albicans *(57.7 ± 3.9% at 30 min and 47.8 ± 3.2% at 120 min, Figure 6A). Elevated rates of salivary neutrophil from aged (74.5 ± 5.7% and 77.4 ± 4.6% at 30 and 120 min) or young patients with D.S. showed viable phagocytosed blastoconidia at both experimental periods (77.4 ± 5.3% and 65.5 ± 6.2%, at 30 and 120 min, respectively, Figure 6A). Neutrophils from aged controls, in opposite to the younger ones, presented significant higher percentages of viable ingested *C. albicans *at 30 and 120 min (79.8 ± 2.3% and 66 ± 4.7%, sequentially, Figure 6A). These results confirm a deficient phagocytosis of *C. albicans *by salivary neutrophils from patients with D.S. In addition, our data showed that elderly people presented neutrophils with impaired ability to neutralize phagocytosed *C. albicans*.

Interestingly, blood neutrophils killed more *C. albicans *than salivary cells considering all the analyzed groups. The percentage mean of blood neutrophils from aged D.S. individuals that kept internalized viable yeasts was higher, but not statistically significant, than that found in the aged control at 30 min (70.3 ± 3.8% and 57.9 ± 5.7%, respectively, Figure 6B). At 120 min, it was observed a reduced difference between aged groups (57.8 ± 2.8% – D.S. and 45.7 ± 5.3%-Control, Figure 6B). Neutralization of *C. albicans *by blood neutrophils from young D.S. individuals was lower in comparison to matched controls since decreased percentages of such cells from young controls did not kill *C. albicans *at 30 and 120 min (27 ± 4.3% and 21.6 ± 3.5% of neutrophils containing viable phagocytosed yeast cells, respectively, Figure 6B).

In summary, there was no statistically significant difference in candidacidal activity between aged groups at any time of the assay. Moreover, blood neutrophils from elderly presenting or not D.S. and from young subjects presenting D.S. neutralized significantly fewer ingested blastoconidia than those from healthy young individuals at both times of evaluation. Photomicrographs 6C and 6D show the viable and dead ingested *C. albicans *inside blood neutrophils by fluorescence assay.

## Discussion

In this report, we provide the first evidence that the number of neutrophils obtained from the saliva of aged or young patients with denture stomatitis is much lower than the number found in the controls. In spite of the lower number of neutrophils that had been obtained from the saliva of the patients, our results showed that these individuals had significantly more circulating neutrophils than controls, probably due to oral infection. Salivary neutrophils arrive in the oral cavity attracted by chemotactic factors via crevicular fluid and salivary glands [18,37-39]. After maxillary teeth loss and consequent partial loss of crevicular fluid, a small number of blood neutrophils would migrate into the oral cavity, and this could be related to a predisposition to denture stomatitis. However, only the absence of maxillary teeth does not seem to explain such decreased number of cells, since the inhibition of neutrophil influx to the oral cavity was also evident in elderly controls compared with that in younger adult controls. This finding shows that alterations in the immune response mechanisms of elderly people (immunosenescence) might be affecting, at least, two distinct steps in neutrophil recruitment to the oral cavity; neutrophil chemotaxis from the circulation to the mouth, and transmigration of these cells across the epithelium into the oral cavity.

Our data showed significant lower expression of CXCR1, CD62L and CD11b on blood neutrophils from elderly, regardless of D.S. These molecules play important role in the tethering, rolling, diapedesis and transmigration of neutrophils responding to chemotactic stimulus [40-43]. The smaller numbers of salivary neutrophils in the oral cavity probably occur, in part, due to the lower CXCR1 expression on blood neutrophils from aged patients and controls. In this way, the neutrophils from aged subjects would not respond to high levels of CXCL8 found in their saliva, culminating in a low number of these cells in the site of stimulus.

We also detected a lower expression of CD62L and CD11b in neutrophils from both elderly groups and young subjects presenting D.S. CD62L shedding from the cell surface of activated granulocytes in the elderly has been observed by other authors [44]. CXCR1 and CD62L reduction could greatly impair Mac-1 function in these leukocytes. In spite of possible alterations caused by *Candida *infection, these data can indicate that ageing leads to a significant reduction in the arrival of neutrophils in response to chemotactic events. Besides, CD11b executes the phagocytic clearance of pathogens [45]. These facts, in turn, might facilitate oral infections in these people. Equally, alterations in the production of neutrophils by the bone marrow over time could be excluded, since there were no differences in the number of blood neutrophils between aged and young subjects evaluated in this study.

Although a possible cytotoxic role of *C. albicans *influencing neutrophils survival and cell number could be considered [46-48] because young and elderly D.S. patients presented a reduced number of salivary neutrophils, such cells were as viable as the salivary neutrophils from the respective controls. Our data indicate that elderly present an elevated rate of apoptotic neutrophils in saliva, regardless of oral infection. This may account for the lower number of neutrophils in saliva from aged persons. The increased apoptosis rates of neutrophils from elderly have been largely described [35]. In this way, the antigenic load in the human saliva could be influencing the lifetime of salivary neutrophils in the elderly in a different manner, in comparison to young individuals. Other authors have reported different results for the elevated rate of apoptosis in blood neutrophils from aged individuals compared to young people after stimulation with cytokines [31]. This prompted us to investigate the cytokines and chemokines present in the saliva, which could be influencing the chemotaxis and survival of neutrophils in the oral cavity.

The data obtained demonstrated that aged individuals have lower salivary levels of GM-CSF, regardless of the disease. The higher level of GM-CSF in the saliva from young D.S. individuals could be protecting their salivary neutrophils from suffering apoptosis induced by *C. albicans *when compared to the respective elderly group. Low levels of GM-CSF in the oral environment might be an important factor predisposing aged individuals to establishment of oral infection since this growth factor plays a fundamental role in the survival and activation of neutrophils [49-52]. In contrast, CXCL8 was detected at drastically elevated levels in the saliva from elderly (patients and controls) in comparison to the respective younger individuals. Further, D.S. aged patients had significantly higher rate of CXCL8 than matched controls. Such difference was not seen in young groups, with or without D.S. Besides, we observed higher levels of TNF-α in the saliva of elderly D.S. than their matched control or young D.S. group. On the other hand, elderly controls had significantly lower saliva levels of TNF-α than younger controls. Probably, the higher level of TNF-α and CXCL8 detected in the saliva from elderly D.S. helps to avoid a more invasive *C. albicans *infection since these cytokines/chemokines play an important role in the innate resistance to oral and systemic *Candida *infections [53,54]. In addition, superior production of TNF-α and CXCL8 by different cells from aged donors has been extensively described [35].

We also provide the preliminary evidence that salivary and blood neutrophils from elderly patients with *Candida*-related denture stomatitis present *in vitro *functional alterations, specifically in the phagocytosis and killing of *C. albicans*. Besides, our results revealed a functional deficiency in the neutrophils associated with age advancement. Although neutrophils phagocytosis could have been evaluated by flow cytometry, we selected the confocal microscopy method because we wanted to guarantee that the complete ingestion of *C. albicans *by neutrophils had occurred. Furthermore, it allowed us to observe the neutralization of ingested blastopores in the same cell samples.

The phagocytic activity of salivary neutrophils from both aged and young D.S. individuals was impaired in comparison to their respective control groups. Furthermore, salivary neutrophils from elderly control group presented reduced phagocytosis in comparison to the matched younger group. Despite of alterations associated with a local influence of fungi, these results indicate that a significant decrease in the phagocytic activity of salivary neutrophils occur in elderly subjects. Regarding the blood cells, the neutrophils from elderly groups (D.S. and control) also presented impaired phagocytic activity when compared to the young groups. The percentage of blood neutrophils phagocytosing *C. albicans *from the young individuals presenting denture stomatitis was not diminished, thus indicating that the alterations in these blood cells appear to be associated with the elderly but not with the occurrence of D.S. Our results are in accordance to previous studies that has broadly reported an age-related reduction in neutrophil phagocytosis [29,35]. Thus, our results agree with the hypothesis that ageing reduces the ability of neutrophils to exterminate *C. albicans *following a pattern that is observed in people infected by this pathogen. The influence of ageing in the neutrophils mechanisms to eliminate *C. albicans *had been previously observed [29]. Altogether, these observations reinforce the idea that elderly are more susceptible to *Candida*-related denture stomatitis than young individuals. Yet, our data suggest that individual predispositions to *Candida*-related denture stomatitis can ocuur, at least relative to the defense mechanisms driven by neutrophils.

## Conclusion

Our results demonstrate that aged and young D.S. individuals show a lower number of salivary neutrophils than controls and present dysfunctions in the phagocytosis and killing of *C. albicans *by both local and circulating neutrophils. Such alterations seem to be more pronounced in the elderly than young adults since aged subjects not infected by *C. albicans *presented impaired neutrophil function. The age advancement appears to influence the neutrophils survival mechanisms even in the absence of the disease. Yet, aged individuals showed low level of GM-CSF and elevated level of CXCL8 in the saliva. A dramatic augmentation of TNF-α was verified in elderly D.S., indicating an exacerbated proinflammatory response against *Candida *infection. Blood granulocytes from elderly with or without *Candida*-related denture stomatitis had a reduced expression of CXCR1, CD62L and CD11b as well as from young patients, which might impair chemotaxis and diapedesis of such cells. Further studies are necessary to fully explore the potential impact of *Candida*-related denture stomatitis in the immune response of neutrophils in the elderly.

## Methods

### Study population

After obtaining the informed consent of the individual in compliance with resolution 196/96 of the National Council of Health and of the University of São Paulo (Bauru, São Paulo, Brazil) committee guidelines, the patients were drawn from the patient pool at the Bauru Dental School Prosthodontics Clinic (USP). Their selection was based on the inclusion and exclusion criteria described below. The exclusion criteria comprised patients suffering from the following conditions: diabetes mellitus, alcoholism, tobacco use, periodontal diseases or other oral pathologies, gingival bleeding, cancer and symptoms that could indicate cancer such as sudden changes in weight and appetite, immune/endocrine/hematological alterations and use of xerostomic, antifungal or antibiotic medications. The inclusion criteria comprised aged subjects presenting *Candida*-related denture stomatitis, which was confirmed by microbiological diagnosis (elderly D.S., *n *= 14) or healthy subjects (elderly control, *n *= 14), ranging from 60 to 85 years old (mean age = 69.4 ± 3 years old in D.S. and 68.6 ± 0.9 years old in control groups). Additionally, individuals ranging from 20 to 50 years old presenting *Candida*-related denture stomatitis (young D.S., *n *= 14, mean age = 33.8 ± 2.3 years) or not (young control, *n *= 14, mean age = 38.1 ± 3.9 years), were assessed. Age limits were determined based on the study of Wenisch *et al. *[29]. In the sampling, all patients with *Candida*-related denture stomatitis were wearers of complete maxillary dentures for at least a two-year period, without natural maxillary teeth. Complete medical and dental histories were recorded. Participants that presented infectious diseases up to one month before saliva sampling, as well as active dental abscesses and collagen vascular diseases, were excluded from the study. None of the *Candida*-related denture stomatitis lesions had been treated in any manner prior to sample collection. None of the control volunteers presented oral lesions.

### Identification of *Candida*-related denture stomatitis

Denture stomatitis was clinically diagnosed and classified according to Newton [55]. One elderly patient presented type III denture stomatitis (granular surface or inflammatory papillary hyperplasia of the palate) and the others showed type I denture stomatitis (pinpoint hyperemia or localized simple inflammation). After such classification, the microbiologic diagnosis was made by collecting material from the hard palate (in a denture stomatitis-associated erythematous area) with a sterile swab and then inoculating it in Sabouraud dextrose broth (Difco, Becton Dickinson, France) with 1% chloramphenicol (0.1 mg/mL, Sigma-Aldrich, St. Louis, USA) at room temperature. After one week, 0.1 mL from broth + sample was spread over Sabouraud dextrose agar plates with 1% chloramphenicol (0.1 mg/mL) to observe colony forming units (CFU). The plates were incubated for 48 h at 37°C.

### Salivary Cytokines

Participants were advised to avoid eating, drinking, using chewing gum, mint, and brushing their teeth for at least two hours before collection. The samples were obtained by requesting subjects to swallow first, tilt their head forward, and then expectorate all saliva into 50 mL centrifuge tubes for 5 consecutive min without swallowing it. Nonstimulated flowing whole saliva samples were collected between 7:00 and 9:00 a.m. in sterilized tubes and centrifuged at 10,000 × g/20 min/4°C. The supernatants of saliva samples were frozen at -70°C until analysis. Interleukin-8 (IL-8, CXCL8), Granulocyte macrophage-colony stimulating factor (GM-CSF) and Tumoral Necrosis Factor-α (TNF-α) were quantified in saliva samples by the quantitative sandwich Enzyme-Linked Immunosorbent Assay (ELISA) using commercial capture and biotinylated detection antibodies (BD Pharmingen Corp., San Diego, CA) and the respective human recombinant cytokines (diluted in PBS) as standards, according to the manufacturer's instructions (cat.#555244 – IL8, # 555126 – GM-CSF, # 555212 – TNF). The concentrations of each cytokine were expressed as pg/mg protein. Previous studies confirmed that cytokines are stable in saliva [56].

### Protein Dosage

Saliva protein content was quantified with Quick Start™ Bradford Protein assay kit (Bio-Rad, CA, USA) and the results expressed in mg/ml. Saliva from elderly stomatitis-positive and stomatitis-negative groups presented protein concentrations of 107.4 and 99.1 mg/ml, respectively. Young groups presented mean values of 117.9 mg/mL (D.S.) and 129.3 mg/mL (controls).

### Antibodies and flow cytometry analysis

For immunostaining of purified blood neutrophils, phycoerithrin (PE) and fluorescein isothiocyanate (FITC) conjugated antibodies against human CXCR1 (CD181, cat. # 555939), CD62L (L-selectin, cat. # 555544) and CD11b (Mac-1, cat. # 555388), and the respective mouse isotype controls were used (BD Biosciences, San Diego, CA). Briefly, 1 × 10^7 ^neutrophils were incubated in 20 μL of each antibody solution for 30 min at 4°C. After washing, the cells in 50 mL of phosphate-buffered saline solution were fixed by 1% paraformaldehyde, and then the binding of each antibody was detected using a flow cytometer. Cell acquisition (counting) was performed on a FACSort flow cytometer using the CellQuest software (BD Biosciences). The mean of fluorescence intensity was determined for each molecule in 30,000 events.

### Isolation of salivary and blood neutrophils

Salivary neutrophils were collected by a modified method described by Lukäc *et al. *[21]. Briefly, volunteers and patients swished 10 mL of sterile 1.5% NaCl solution without swallowing it for 4 min and expectorated into a sterile beaker. This procedure was further repeated four times. Denture wearers were oriented to take their prosthesis off before the collection. After that, the collected material was centrifuged at 450 × *g*/10 min. The supernatant was discharged and the pellet suspended in 2 mL of RPMI 1640 medium containing 10% of fetal calf serum (10% FCS RPMI) (Gibco, Invitrogen, UK) including 1 mM sodium pyruvate, 0.1 mM nonessential amino acids, 30 mM HEPES, 100 U/mL penicillin, 0.1 mg/mL streptomycin. Samples were gently vortexed in order to avoid aggregation of neutrophils and the cell suspensions were sequentially filtered through 20 and 11 μm nylon filters (NY2002500 and 1102500, MilliUni, Millipore Corporation). The filtered suspension was washed once at 250 × *g*/10 min, the supernatant was discharged and the pellet was suspended in 1 mL of 10% FCS RPMI medium.

Heparinized whole blood (10 mL) was obtained from volunteers and patients by venipuncture, and blood neutrophils were purified as follows. Briefly, 5 mL of Histopaque 1119 (Sigma-Aldrich Brazil Ltda., São Paulo, Brazil) were poured into a 15 mL round bottom tube and overlaid with 3 mL of Histopaque 1083 (Sigma-Aldrich Brazil Ltda., São Paulo, Brazil), and 6 mL of whole blood were layered over the gradients. Tubes containing gradients and blood were centrifuged (460 × *g*) for 28 min at 25°C. The second band, which contained the neutrophils, was aspirated and washed twice with cold RPMI 1640.

The salivary and blood (systemic) neutrophils were identified through staining with Türk's solution. The viability of salivary and blood neutrophils was analyzed by positivity for annexin V-FITC or propidium iodide (Aposcreen Annexin V-FITC, R&D Systems, Minneapolis, USA). The cells were analyzed by fluorescence microscopy using a Axiostar plus HBO 50/AC (Zeiss, Germany) and the percentage of apoptotic cells was calculated from the proportion of positive neutrophils (green and/or red cells) in relation to total neutrophils [46].

### *Candida albicans *ATCC 10231

*C. albicans *ATCC 10231 strains was obtained from the stock culture collection of the Department of Immunology and Microbiology, Bauru School of Dentistry, University of São Paulo. Yeast cells were grown on Sabouraud dextrose Agar and maintained at 28°C. Before infection, the blastoconidia were grown in Sabouraud dextrose broth for 48 h at room temperature. At the moment of the opsonization assay, these yeast cells were harvested by centrifugation (500 × *g*/6 min), washed and suspended at the desired concentration in Phosphate Buffered Saline (PBS).

### Opsonization of *C. albicans*

Blastospores were opsonized in 1 mL of human serum during 30 min/37°C/5% CO_2_, washed twice with PBS, and the pellet was suspended in PBS and maintained at 4°C until the procedures were carried out [57].

### Phagocytosis and Killing Assay

Blood or salivary neutrophils were cultured with viable *C. albicans *(at a ratio of 1:1 – opsonized yeast: viable neutrophil) in 10% FCS RPMI at 37°C/5% CO_2_. After 30 and 120 min, the cell collection was centrifuged (350 × *g*/10 min) and the pellet was suspended in PBS. Neutrophils phagocytosing opsonized yeast forms of *C. albicans *were distributed on coverslips and analyzed after staining with 0.05 mg/mL of acridine orange solution in PBS and visualized with UV light under the fluorescence microscope Axiostar plus HBO 50/AC (Zeiss, Germany) and photographed by confocal laser scanning microscopy (TCS model, SPE, Leica, Mannheim, Germany).

Neutrophils phagocytosing or not *C. albicans *(showing green or orange colors, which indicate viable and dead blastospores, respectively) were counted [29]. The results of the phagocytic activity as well as of the internalized fungal cell viability were expressed as the mean percentage of viable neutrophils containing at least one yeast cell by random counting of 10 fields. All assays were prepared and analyzed in duplicate.

### Statistical Analysis and Software Packages

Statistical analysis was performed using GRAPHPAD INSTAT version 2.04a. All results were analyzed through nonparametric tests. One-way ANOVA followed by Tukey's test was employed and significant differences were considered for *P *< 0.05. GraphPad PRISM (version 4) was used for graphic representations. To compare differences between two groups unpaired t-test followed Mann-Whitney test was applied and significant differences were considered for *P *< 0.05.

## Abbreviations

D.S: *Candida *related-denture stomatitis.

## Competing interests

The authors declare that they have no competing interests.

## Authors' contributions

THG had the overall responsibilities of the experiment design and statistical analysis, and wrote the manuscript. NAV assisted to conduct the samples collection. VCP carried out the clinical diagnosis and volunteers/patients selection. APC and VSL had shared the concept and supported the manuscript. VSL had overall responsibilities of fund management, experimental design and wrote the manuscript. All the authors have read and approved the final manuscript.
